# Heavy metals content in ashes of wood pellets and the health risk assessment related to their presence in the environment

**DOI:** 10.1038/s41598-021-97305-4

**Published:** 2021-09-09

**Authors:** Mirha Pazalja, Mirsada Salihović, Jasmina Sulejmanović, Alisa Smajović, Sabina Begić, Selma Špirtović-Halilović, Farooq Sher

**Affiliations:** 1 University of Sarajevo-Faculty of Pharmacy, Zmaja od Bosne 8, 71000 Sarajevo, Bosnia and Herzegovina; 2 University of Sarajevo-Faculty of Science, Zmaja od Bosne 33-35, 71000 Sarajevo, Bosnia and Herzegovina; 3 School of Science and Technology, Nottingham Trent University, Nottingham, NG11 8NS UK

**Keywords:** Ecology, Environmental social sciences

## Abstract

Efforts to reduce air pollution in developing countries may require increased use of biomass fuels. Even biomass fuels are a sustainable alternative to fossil fuels there is limited quantitative information concerning heavy metal content in their ashes. Therefore, this study focuses on the determination of the heavy metal concentrations in wood pellet ash obtained from the combustion of 10 pellet brans from Bosnia and Herzegovina and Italy, the effects of adding the ashes to soils, and the assessment of health risk assessment. Ash content was determined by gravimetric method. The amount and composition of ash remaining after combustion of wood pellets varies considerably according to the type of biomass and wood from which the pellet is made. Samples were prepared by wet digestion using HNO_3_, and heavy metals are determined by atomic absorption spectroscopy-flame and graphite furnace. The results showed that the lowest concentration in ashes was obtained for Co 0.01 mg kg^−1^ and the highest for Fe 571.63 mg kg^−1^. The Hazard Index (HI), calculated for non-cancerous substances for children was 2.23E−01, and the total Risk index was 4.54E−05. As for adults, HI was 1.51E−02, while the Risk index value was 3.21E−06. Human health risk calculated through HI and Risk index for children and adults associated with analyzed pellets is not of significant concern. The calculated enrichment factor and metal pollution index for wood pellet ashes indicate the risk of soil contamination with heavy metals. From this point of view, analyzed samples of ashes could be a serious contaminant of soil, so further monitoring is required.

## Introduction

The energy production from wood biomass fuels is a sustainable and environmentally friendly alternative to the use of fossil fuels. Biomass refers to any material produced by organisms of animal and plant origins, such as wood, agricultural residues, and animal residues, which can be used as fuel for energy production^1^. Wood as an energy source is increasingly returning to use and wood pellets as one of the most common solid biomass wares are specially used for energy intents. Pellet is a cylindrical organic fuel produced by compressing biomass fuel, which usually consists of wood waste, agricultural biomass, merchandisable grasses, and forestry remains^2^. Wood pellets fuel quality as well the efficiency of the devices which capture and store combustion products is of great importance. The incinerating of wood pellets is a simple household available source of heat and energy which generated waste, primarily ash^3^. Ashes are the proportion of small particles of material produced by the combustion of solid fuels like coal, wood, and other high-energy substances. The composition of ashes depends on the type of material burned and the boilers used in the process^4,5^. The ash produced during the burning of wood pellets in households represents an additional and needless problem on the environment due to non-selective collection and landfill disposal. However, the ash produced from wood biomass is possible to apply in agriculture as soil fertilizer because of constituted nutrients and vital inorganic components such as C, O, H, Ca, K and, less frequently, N, S, Mg, P, Cl, Na, Mn, Zn, Fe, B, Cu or Mo. This application improves soil balance by providing a liming effect (alkaline pH)^6,7^. But, the pollution problem caused by ashes disposing is especially serious because they can contain high heavy metal concentrations^8^. Heavy metals are natural elements with relatively high density, atomic numbers, and atomic weight. Their multiple uses in industry, households, agriculture, medicine, and technology raise concerns about their potential impact on human health and the environment^9^. Heavy metals such as Cd, Hg, Pb, Cr, Ni, and As, present even at very low concentrations have harmful effects on the body causing acute and chronic toxicity in humans^10,11^. Therefore, for environmental protection, as well as to provide sufficient clean air and soil levels, heavy metals should be kept at a safe level^12^. This is the main reason why monitoring of different energy intents (i.e. fuel, biomass, wood pellets, coal) should be performed based on analyzing the heavy metal content in their ashes. The house heating in Bosnia and Herzegovina (B&H) is generally based on using solid, conventional fuels. The requirements for using renewable energy sources have increased the contribution of the wood biomass use which results in rises in the quantity of the waste incinerated, primarily in the form of ashes. Many researchers have provided results about the physical and chemical characteristics of wood pellets, but such studies are still lacking for wood pellets used in B&H^13,14^. Additionally, some authors have found high concentrations of some heavy metals in particulate matter found in the air or street dust of B&H. They only propose that wood pellet from household is one of the sources of heavy metals in the ambient air or street dust of B&H, but the main information about heavy metal content in used pellets is missing^15–17^. Considering the above, this study is aimed to determine the heavy metal concentrations in the ashes produced by combusting ten wood pellet samples often used in B&H. Furthermore, the novelty of the work is related to health risk assessment associated with the presence of polluted ashes in the environment for children and adult residents in the region. Obtained results could be used in future work to find out how much wood pellets contribute to the total air pollution.

## Materials and methods

### Collection of the samples

Ten (10) wood pellet samples were purchased from a different location in B&H, of known suppliers from the market (supermarkets, garden shops, and gas stations). The samples were accompanied by a declaration describing that nine of them were originated from B&H, and one of them was from Italy. Characteristics of collected wood pellet samples (type of wood, energetic value, declared moisture, declared and determined ash amount) are listed in Table 1. All of the samples were analyzed for moisture and ash content. Additionally, in ash samples of mentioned wood pellets, heavy metal concentration (Cd, Co, Cr, Cu, Fe, Mn, Ni, Pb, and Zn) was determined.

**Table 1:** Characteristic of analyzed samples wood pellets.

Sample	Energetic value (kWh/kg)	Wood type	Ashes declared (%)	Ashes founded (%)	Moisture declared (%)	Country of origin
S1	5	Beech (80%), oak (20%)	0.64	1.11	5.24	B&H
S2	5.45	Beech, fir	0.60	0.96	10.00	B&H
S3	4.58–5.27	Beech	≤ 0.70	0.71	≤ 10.00	Italy
S4	5.1	^a^Oak, beech, ash tree, hornbeam	0.75	1.56	8.40	B&H
S5	4.88	^a^Oak, beech, ash tree, hornbeam	< 1.20	1.29	< 10.00	B&H
S6	5.20	Spruce (50%), beech (50%)	< 1.00	2.36	< 10.00	B&H
S7	4.83	Coniferous wood	0.40	1.56	7.40	B&H
S8	5.10	Spruce (50%), beech (50%)	< 0.70	1.00	≤ 8.00	B&H
S9	4.60	Beech, spruce, fir	–	1.21	≤ 10.00	B&H
S10	5.20	70% Beech, 30% fir and spruce	< 0.70	2.14	< 10.00	B&H

^a^Wood from forest waste, firewood, sawdust, and other wood processing waste.

All pellet samples were originated from B&H, purchased from different cities, often used for house heating, instead of sample S3 which was from Italy.

### Ash determination of wood biomass samples

The wood pellet samples were oven-dried at 105 °C for 24 h. The content of ash was determined by gravimetric method according to the procedure published by Pan and Eberhardt^18^ as follows: pellet samples, 1 g (± 0.1 mg) of each was weighed into a previously annealed ceramic pot (m_1_) and burned in a muffle furnace (Nabertherm) for one hour at 300 °C, following by increasing the temperature to 400 °C for one hour more and then burning the samples for next six hours at 550 °C. The procedure is repeated until a constant mass (m_2_) was reached. The ash content is determined by the Eq. (1):

1 $${\text{Ash content}}, \% = \frac{{{\text{(m}}_{2} - {\text{m}}_{{1}} {)}}}{{{\text{m}}_{{{\text{sample}}}} }} \times {100 }{\text{.}}$$

### Preparation of samples

The chemical determinations of the heavy metals in wood pellet ashes (Table 2) were made by wet digestion by soaking the samples in 25 mL of 65% HNO_3_ in polytetrafluoroethylene (PTFE) vessels. After evaporation of the nitrogen oxides, the vessels were closed and allowed to react for 14 h at 80 °C, following by cooling to room temperature. Then, the digest was filtered, transferred to a 25 mL volumetric flask, and filled up with redistilled water to the mark. All samples and blank were prepared in three replicates^19–21^.

**Table 2:** Heavy metal concentrations (mg kg^−1^ d.w.) in the wood pellet ashes.

Metals	S1	S2	S3	S4	S5	S6	S7	S8	S9	S10	Mean
Ash masses (mg)	11.10	9.60	7.10	15.60	12.90	23.60	15.60	10.00	12.10	21.40	–
Cd	1.02 ± 0.03	0.50 ± 0.09	0.11 ± 0.01	0.75 ± 0.03	0.70 ± 0.02	0.96 ± 0.04	0.28 ± 0.14	0.30 ± 0.11	0.40 ± 0.17	0.58 ± 0.14	0.56
Co	0.05 ± 0.03	0.04 ± 0	0.16 ± 0.01	0.17 ± 0.13	0.84 ± 0.16	0.19 ± 011	0.03 ± 0.01	0.01 ± 0	0.58 ± 0.18	0.02 ± 0	0.21
Cr	0.66 ± 0.26	0.29 ± 0.14	1.11 ± 0.19	1.26 ± 0.14	2.04 ± 0.20	2.71 ± 0.16	0.15 ± 0.01	0.13 ± 0.02	0.28 ± 0.11	0.10 ± 0	0.87
Cu	3.38 ± 0.38	2.38 ± 0.23	1.63 ± 0.11	2.13 ± 0.25	1.25 ± 0.17	2.25 ± 0.25	9.95 ± 0.54	2.25 ± 0.11	9.15 ± 0.42	4.43 ± 0.27	3.88
Fe	31.25 ± 0.50	33.63 ± 0.38	34.38 ± 0.88	88.13 ± 2.38	190.00 ± 10.00	250.00 ± 5.00	571.63 ± 10.11	240.48 ± 10.53	327.70 ± 10.14	236.15 ± 3.00	200.34
Mn	34.88 ± 0.63	38.38 ± 0.52	52.00 ± 0.75	110.50 ± 6.25	47.13 ± 1.88	64.50 ± 0.75	32.53 ± 0.32	19.60 ± 0.28	20.23 ± 0.32	24.50 ± 0.28	44.43
Ni	1.35 ± 0.18	0.66 ± 0.11	1.49 ± 0.19	1.11 ± 0.13	1.39 ± 0.21	1.44 ± 0.25	0.90 ± 0.12	0.63 ± 0.13	2.63 ± 0.26	0.20–0.05	1.18
Pb	0.56 ± 0.10	0.43 ± 0.15	0.67 ± 0.21	0.24 ± 0.14	0.31 ± 0.16	0.71 ± 0.25	3.65 ± 0.40	2.50 ± 0.22	6.83 ± 0.39	8.08 ± 0.55	2.40
Zn	7.24 ± 0.16	11.74 ± 0.04	8.71 ± 0.56	12.84 ± 0.21	5.83 ± 0.20	8.30 ± 0.30	19.05 ± 1.30	6.38 ± 0.46	25.83 ± 2.56	19.08 ± 2.54	12.50
Total	80.39	88.05	100.26	217.13	249.49	331.06	638.17	272.28	393.63	293.14	266.36

### Heavy metal analysis

Metal analyses in ash samples of mentioned wood pellets were performed using a flame atomic absorption spectrometry (Varian AA240FS) for Mn, Fe, Pb, and Zn and graphite furnace (Varian AA240Z) for Cd, Co, Cr, Cu, and Ni. A blank probe was prepared using the same digestion method to avoid the matrix effect. Standard metal solutions used for the calibration graphs were prepared by diluting 1000 mg L^−1^ stock single-element atomic absorption standard solutions of Cd, Co, Cr, Cu, Fe, Mn, Ni, Pb, and Zn (Certipur Grade, Merck, Germany). Linear calibration graphs with correlation coefficients > 0.99 were obtained for all analyzed metals. The accuracy of the method was evaluated using the standard reference materials: Fine Fly Ash (CTA-FFA-1, Institute of Nuclear Chemistry and Technology Poland) and Fly Ash from pulverized coal (BCR-038, Institute of reference materials and measurements-IRMM, Belgium). The obtained results were in the range of the reference materials. The detection limit (LOD) and limit of quantification (LOQ) for the nine analyzed metals were calculated based on X_b_ + 3 SD_b_ and X_b_ + 10 SD_b_, respectively, where X_b_ is the mean concentration of the blank sample (n = 8) and SD_b_ is the standard deviation of the blank for eight readings^22^. The values of the LOD were: Cd (0.61 µg L^−1^), Co (0.49 µg L^−1^), Cr (0.67 µg L^−1^), Cu (20.10 µg L^−1^), Fe (83.85 µg L^−1^), Mn (6.42 µg L^−1^), Ni (1.12 µg L^−1^), Pb (23.77 µg L^−1^), Zn (58.68 µg L^−1^), and LOQ values were: Cd (1.25 µg L^−1^), Co (1.41 µg L^−1^), Cr (1.42 µg L^−1^), Cu (47.66 µg L^−1^), Fe (111.2 µg L^−1^), Mn (16.14 µg L^−1^), Ni (2.70 µg L^−1^), Pb (47.73 µg L^−1^) and Zn (71.05 µg L^−1^).

### Pollution evaluation

The metal pollution index (MPI) as the geometric mean of the concentration of all metals found in ashes of wood samples was calculated by the following Eq. (2)^23^:

2 $${\text{MPI}} = \left( {{\text{C}}_{1} \cdot {\text{C}}_{2} \cdot \cdots {\text{C}}_{{\text{k}}} } \right)^{{1/{\text{k}}}} ,$$

where C_1_ is the concentration value of the first metal, C_2_ is the concentration value of the second metal, C_k_ is the concentration value of the kth metal.

Evaluation of the presence and the grade of anthropogenic activity were demonstrated through the calculation of the enrichment factor (EF), widely used in environmental issues^24^. To understand which elements were relatively enriched in the different wood pellet ash samples, the heavy metal enrichment factor was calculated relative to soil values according to Eq. (3)^25^.

3 $${\text{EF}} = \frac{{\left( {\frac{{{\text{C}}_{{\text{k}}} }}{{{\text{E}}_{{{\text{ref}}}} }}} \right)_{{{\text{ashes}}}} }}{{\left( {\frac{{{\text{C}}_{{\text{k}}} }}{{{\text{E}}_{{{\text{ref}}}} }}} \right)_{{{\text{soil}}}} }},$$

where C_k_ is the concentration of the element in the sample or the soil, E_ref_ the concentration of the reference element used for normalization. A reference element is an element commonly stable in the soil characterized by the absence of vertical mobility and/or degradation phenomena. As in many studies as a reference element were Fe, Al, Mn, Sc, or total organic carbon used^26,27^. Therefore Fe has been chosen as reference material in this study. Iron is one of the major constituents of soil, as well as the average chemical constituent of the upper continental crust^26^.

### Health risk assessment

The general exposure equations used in this study were adapted according to the US Environmental Protection Agency guidance^28–30^. The daily exposure (D) to heavy metals via wood pellet ash was calculated for the three main routes of exposure: (i) direct ingestion of ash particles (D_ing_); (ii) inhalation of suspended particles via mouth and nose (D_inh_); and (iii) dermal absorption to skin adhered ash particles (D_dermal_). Equations (4) to (6) were used to calculate exposure via ingestion, inhalation, and dermal route, respectively^22,31^.

4 $${\text{D}}_{{{\text{ing}}}} = {\text{ C }} \cdot \frac{{{\text{ IngR }} \cdot {\text{ EF }} \cdot {\text{ ED}}}}{{{\text{BW }} \cdot {\text{ AT}}}}{ } \cdot {\text{CF}}1{, }$$

5 $${\text{D}}_{{{\text{inh}}}} = {\text{ C }} \cdot \frac{{{\text{ InhR}} \cdot {\text{ EF }} \cdot {\text{ ED}}}}{{{\text{PEF }} \cdot {\text{ BW }} \cdot {\text{ AT}}}}{, }$$

6 $${\text{D}}_{{{\text{dermal}}}} = {\text{ C }} \cdot \frac{{{\text{ SA }} \cdot {\text{ SL }} \cdot {\text{ABS }} \cdot {\text{EF }} \cdot {\text{ ED}}}}{{{\text{BW }} \cdot {\text{ AT}}}}{ } \cdot {\text{CF}}1{, }$$

where c (mg kg^−1^) is the heavy metals concentrations in ash samples; IngR (mg day^−1^) is the conservative estimates of dust ingestion rates, 50 for adults, 200 for children^30,32^; InhR (m^3^ h^−1^) is the inhalation rate, 2.15 for adults, 1.68 for children^32^; EF (h year^−1^) is the exposure frequency, 1225 for adults and children^22^; ED (years) is the exposure duration, 70 for adults, 6 for children^22^; BW (kg) is the body weight, 80 for adults, 18.60 for children^32^; AT (days) is the averaging time, 25,550 for adults, 2190 for children^22^; PEF is the particle emission factor (m^3^ kg^−1^), 6.80 × 10^8^ for adults and children^31^; SA (cm^3^) is the exposed skin area, 6840 for adults, 2550 for children^32^; SL (mg cm^−2^) is the skin adherence factor, 0.22 for adults, 0.27 for children^32^; ABS is the dermal absorption factor, 0.001 for adults and children^31^; CF1 is the unit conversation factor, 10^–6^ for adults and children^22^.

The potential non-carcinogenic risk for each metal was estimated using the Hazard coefficient (HQ), as suggested by US EPA^33^. The HQ under various routes of exposure such as ingestion (HQ_ing_), inhalation (HQ_inh_), and dermal (HQ_dermal_) was calculated as a ratio of daily exposure (D) to reference dose of each metal (RfD) according to Eq. (7)^32^.

7 $${\text{HQ}}_{{\text{k}}} = \frac{{{\text{D}}_{{\text{k}}} }}{{{\text{RfD}}}},$$

where k is ingestion, inhalation, or dermal route. The total hazard index (HI) of heavy metal for all routes of exposure was calculated as a sum of HQ_ing_, HQ_inh_, and HQ_dermal_ as given in Eq. (8)^34^.

8 $${\text{HI}} = {\text{ HQ}}_{{\text{ing }}} + {\text{ HQ}}_{{\text{inh }}} + {\text{ HQ}}_{{\text{dermal }}} .$$

The carcinogenic risk (Risk) for potential carcinogenic metals was calculated by multiplying the doses by the corresponding slope factor (SF), as given in Eq. (9)^35^. The carcinogenic oral, inhalation, and dermal SF, as well as dermal absorption toxicity values, were provided from the Integrated Risk Information System^30^. The reference doses for Pb were taken from the Guidelines for Drinking Water Quality published by the World Health Organization^36^.

9 $${\text{Risk}} = { }\mathop \sum \limits_{{{\text{k}} = 1}}^{{\text{n}}} {\text{D}}_{{\text{k}}} \cdot {\text{ SF}}_{{\text{k}}} ,$$

where SF is the cancer slope factor for individually metal and k route of exposure (ingestion, inhalation, or dermal path). The total cancer risk (Risk_total_) of potential carcinogens was calculated as the sum of the individual risk values using the following Eq. (10).

10 $${\text{Risk}}_{{{\text{total}}}} = {\text{Risk}}_{{{\text{ing}}}} + {\text{Risk}}_{{{\text{inh}}}} + {\text{Risk}}_{{{\text{dermal}}}} .$$

## Results and discussions

### Concentration of heavy metals in the ashes of wood pellets

The content of heavy metals in wood pellet ash produced by biomass combustion depends on several factors: the type and quality of wood biomass, the production process, the use of additives, the characteristics of the furnace, the temperature of the process, etc. The results of the heavy metal contents in the wood pellet ash samples represent the mean values of three replicate determination and are given in Table 2. The total concentrations of nine (9) tested metals were expressed as the sum of the metal concentrations in the ash for ten (10) collected wood pellet samples. The total heavy metal concentrations ranged from 80.39 mg kg^−1^ (S1) to 638.17 mg kg^−1^ (S7). The mean concentrations of analyzed heavy metals decrease as follows Fe > Mn > Zn > Cu > Pb > Ni > Cr > Cd > Co.

For comparison, the literature values of heavy metal concentrations in wood ash and ash of different wood biomass regarding the extraction procedure are presented in Table 3. The total heavy metal contents obtained after HNO_3_ extraction of wood samples are comparable to the results presented by other authors, especially for Cd, Cu, and Pb^13,37^. Considerable higher concentrations of Cr, Cu, Ni, and Zn in ash were found by Eberhardt and Pan^14^. However, because of various sample preparation methods used, different wood composition, and dissimilar combustion methods used, the studied ash shows variety in the heavy metal contents. This diversity in chemical composition is crucial in the finding of possible ways of utilizing ash for dispersion into the soil or use for other purposes^5,38^. European legislation of the ash utilization in forestry and agriculture is diverse in different countries. In all analyzed samples, metal concentrations are lower than the limit values for some European countries such as Germany, Sweden, and Denmark^39^.

**Table 3:** The total heavy metal concentration (mg kg^−1^) in wood ash by different authors regarding the method of extraction.

Methods of extraction	Cd	Co	Cr	Cu	Mn	Ni	Pb	Zn	References
HNO_3_ and HCl (3:1)	< 0.30	2.50	15	< 10	–	19	< 3	160	^13^
HNO_3_ and HCl (1:3)	4.39	0.50	38.50	37.20	–	47.30	11.80	345	^14^
HNO_3_	4.54	0.70	37.50	37.80	–	47.50	12.90	357	^14^
–	0.40–0.70	0–7	> 60	15–300	2–5.50	40–250	15–60	15–103	^37^
HNO_3_	0.11–1.02	0.01–0.84	0.10–2.71	1.5–9.95	19.60–110.50	0.20–2.63	0.24–8.08	5.83–19.08	This study

''–'' no data; bottom ash of wood chips, sawdust, bark, and peat^13^; flay ash of wood chips^14^; bottom wood ash^37^; bottom wood pellet ash (this study).

Additionally, a more comprehensive comparison in the meaning of detailed differences between the minimum and maximum values of heavy metals were compared to similar investigations considering the combination of biomass (same or similar origin) and the same combustion temperature to ashes (500–600 °C) are shown in Tables 4 and 5. Analyzing the results from Tables 4 and 5, it could be concluded that the ash contribution of the studied biomass used often in B&H is very low, which is typical for given raw material.

**Table 4:** Comparison of obtained values of heavy metals (Cd, Co, Cr, and Cu) in analyzed wood pellets with similar studies.

Origin/type	Ash (%)	Concentration mg kg^−1^					References
		Value	Cd	Co	Cr	Cu	
–/oak	2.21	Min	5.92	–	22.41	304.0	^8^
		Max	8.26		34.92	307.0	
Lublin/beech, hornbeam	10.18	Min	–	–	12.49	25.21	^40^
		Max			20.65	104.00	
Italy/beech, conifers	< 0.70	Min	0.08	–	0.46	2.20	^25^
		max	1.00		2.20	4.40	
Canada/fir	< 0.40	Min	0.08		0.93	4.60	^25^
		Max	0.10		3.40	8.50	
Finland/–		Mean	–	–	–	23	^41^
B&H/beech, fir, oak, hornbeam, spruce	1.47	Min	0.11	0.01	0.10	1.25	This study
		Max	1.02	0.84	2.71	9.95

**Table 5:** Comparison of obtained values of heavy metals (Fe, Mn, Ni, Pb, and Zn) in analyzed pellets with similar studies.

Origin/type	Ash (%)	Concentration mg kg^−1^						References
		Value	Fe	Mn	Ni	Pb	Zn	
–/oak	2.21	Min	4271	–	16.40	9.36	616.67	^40^
		Max	5390		16.82	9.69	716.00	
Lublin/beech, hornbeam	10.18	Min	1088	313.33	68.57	9.08	68.83	^8^
		Max	4560	482.67	99.07	11.23	160.33	
Italy/beech, conifers	< 0.70	Min	119.00	60.00	0.33	0.23	2.30	^25^
		max	377.00	68.00	2.60	0.70	7.80	
Canada/fir	< 0.40	Min	83.00	68.00	0.18	0.51	6.70	^25^
		Max	247.00	70.00	1.50	0.56	10.0	
Finland/–		Mean	–	1370	–	–	350	^41^
Bosnia and Herzegovina/beech, fir, oak, hornbeam, spruce	1.47	Min	31.25	19.60	0.20	0.24	5.83	This study
		Max	571.63	110.50	1.49	8.08	25.83	

Generally, the ash content for wood is often less than 2%^42^. However, under incomplete combustion due to unburnt organic material, high values of ash content could be obtained, etc. In the case of biomass from Lublin^8^. Sampling site, harvesting time as well as harvest conditions are significant factors that contribute to the ash content of biomass.

Regarding the heavy metal content in the studied biomass, it could be concluded that the results varied within very wide limits. The highest value for Cd, Cr, Cu, Fe, and Zn was found in the ash biomass of oak, for which the highest content of ash was also recorded^40^. Except for oak ash, high values for Cd, Cr, Cu, and Fe were also recorded for the ash of beech and hornbeam wood type^8^. Furthermore, the highest content of Mn, Ni, and Pb was also obtained for beech and hornbeam wood type^8^, while for Zn, the value corresponds to the wood pellet ash originating from a grate-fired boiler at a small-scale, heating plant at Kuusamo, Eastern Finland^41^. Differences between particular types of organic material, regarding the chemical composition, vary significantly due to different factors i.e.: tree species, growing site, climate and tree component (bark, wood, and leaves), age of the tree, etc. However, detailed analyses and further monitoring of biomass are needed due to insufficient data about the content of hazardous elements in it. The presented results were furthermore compared with the limit value (forest fertilizer) for wood, peat, and biomass-derived ashes used as forest fertilizer to conclude the possible use of such ashes. The limit value of 17.5; 700; 150; 300; 4500; and 150 mg kg^−1^ for Cd, Cu, Pb, Cr, Zn, and Ni is stated as maximal allowable heavy metal concentrations in forest fertilizer^43^. We compared obtained results of heavy metals in ash samples with the limit values given by EU directives and regulations^43^. All analyzed samples have metal concentrations lower than the limit values.

### Metal pollution index

**Figure 1:**
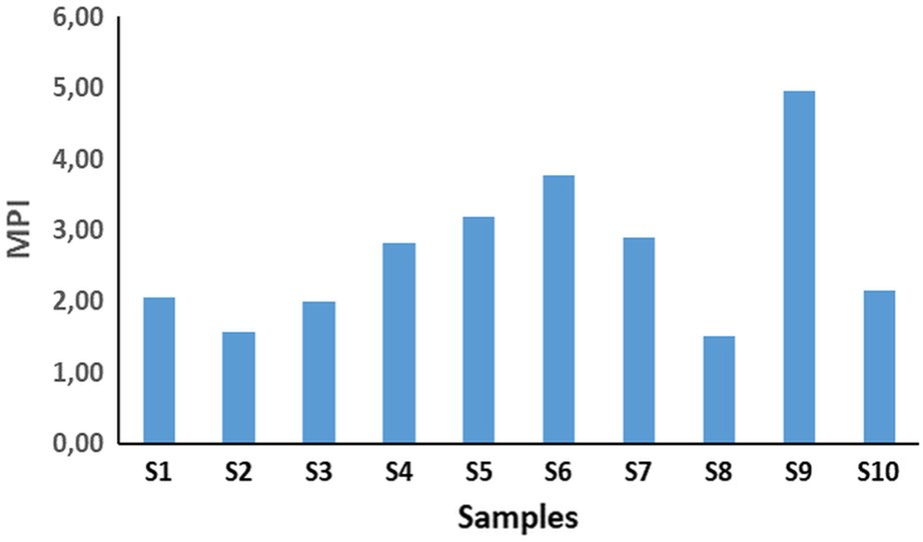
MPI values of analyzed wood pellet ashes.

In addition to the above, to compare the total metal content in analyzed ash samples the MPI was used. MPI is an important and precise way of monitoring metal pollution levels in different contaminated mediums^44^. The obtained results for MPI are presented in Fig. 1. The MPI values in this study ranged from 1.51 to 4.96. The highest MPI value was measured for Sample 9 (beech, spruce, fir), while the lowest one for Sample 8 (beech, spruce). Higher MPI of analyzed samples reflects heavy metal richness in wood pellet ashes, which can cause the accumulation of heavy metals in the soil during ash disposal. Comparing the results of MPI presented in Fig. 1 with a similar study for wood pellet ash from Italy^25^, it could be concluded that the MPI values in this study were lower. Therefore, it could be mentioned that the pollution with heavy metals by using wood pellets described in this work would be less significant than those when using wood pellet samples from Italy as an energy source.

### Enrichment factor (EF)

Moreover, the EF was used to value the effect of the possible addition of wood pellet ashes to soils. An important condition for the maintainable use of ashes in agriculture is the assessment of possible environmental impacts. Numerical values of EF indicate different levels of pollution. Values of EF < 2 suggest the matrices can be classified as a deficiency to minimal enrichment. While, a 2 < EF < 5 indicates moderate enrichment, 5 < EF < 20 significant enrichments, 20 < EF < 40 very high enrichment and EF > 40 extremely high enrichment^45^. Enrichment factors obtained for analyzed samples have values from 0.97 for Co (S6) to 5216 for Cd (S1) (Table 6). The mean EF values for Mn, Cd, Pb, Zn, and Cu are significantly higher than 10, for Co and Cr less than 10, while for Ni it is close to 10. EFs much higher than 10 are considered to initiate primarily originated from anthropogenic sources. Therefore, the metal content in the analyzed wood pellet ash indicates that it is a serious contaminant of soil and the environment.

**Table 6:** Enrichment factors for analyzed samples.

Metals	S1	S2	S3	S4	S5	S6	S7	S8	S9	S10	Mean
Mn	59.37	60.7	80.45	66.7	13.19	13.72	3.04	4.30	3.29	5.53	31.03
Ni	18.46	8.39	18.52	5.38	3.12	2.46	0.67	1.12	3.42	0.36	6.19
Co	2.05	1.52	5.96	2.46	5.66	0.97	0.07	0.02	2.26	0.05	2.10
Cd	5216	2378.82	535.19	1361.62	589.47	614.4	78.37	199.60	195.30	392.97	1156.17
Pb	28.64	20.45	31.18	4.35	2.61	4.54	10.22	16.63	33.35	54.74	20.67
Zn	104.5	157.25	114.12	65.63	13.82	14.95	15.01	11.95	35.51	36.39	56.91
Cu	138.54	90.61	60.71	30.95	8.42	11.52	22.28	11.98	35.74	24.01	43.48
Cr	7.94	3.24	12.14	5.38	4.03	4.08	0.10	0.20	0.32	0.16	3.76

### Non‑carcinogenic and carcinogenic hazards for the ash samples

To assess the impact of heavy metals in wood pellet ashes on children and adults' health or the environment in general Hazard Index HI for non-cancerogenic substances and Risk index for cancerogenic substances was used. The obtained results for HI are presented in Table 7. The calculation was realized for exposure pathways by ingestion, inhalation, and dermal contact. For children, obtained results showed that the total hazard index HI for non-carcinogenic substances was 2.23E−01. Regarding total non-carcinogenic risk for children, it has a value less than 1 (HI < 1), which indicates that there is a very low non-carcinogenic risk for heavy metals in the ash formed by the burning of wood pellets. The highest value for HI was obtained for the ingestion pathway (1.78E−01). Therefore, the ingestion pathway represents the highest risk, followed by dermal contact (9.43E−03), while the inhalation pathway represents the lowest risk (5.95E−06). The contribution of elements to the total HI value for children decrease in the following order: Mn > Co > Cd > Pb > Cr > Cu > Ni > Zn. The highest values obtained for Mn, Co, and Cd are similar to the previous study of wood pellet ashes^25^. For adults, the total HI was 1.51E−02. The results were similar to those obtained for children, as the dominant exposure pathway was ingestion (1.03E−02). The values for dermal contact were lower (4.80E−03), and very low for inhalation (1.77E−06).

**Table 7:** The reference doses, hazard coefficient, and non-carcinogenic hazard index for children and adults.

Metal	RfD (mg kg^−1^ per day)			Children				Adults			
	Ing	Inhal	Dermal	HQ_ing_	HQ_inh_	HQ_der_	HI	HQ_ing_	HQ_inh_	HQ_der_	HI
Mn	1.40E−02	–	–	1.15E−01	–	–	1.15E−01	6.66E−03	–	–	6.66E−03
Ni	2.00E−02	2.00E−03	5.40E−03	2.13E−03	2.63E−07	2.71E−05	2.16E−03	1.24E−04	7.83E−08	1.38E−05	1.38E−04
Co	2.00E−02	3.00E−05	1.60E−02	3.77E−04	3.11E−06	1.62E−06	3.64E−02	2.19E−05	9.24E−07	8.25E−07	2.36E−05
Cd	1.00E−03	1.00E−03	1.00E−05	2.02E−02	2.50E−07	6.96E−03	2.72E−02	1.18E−03	7.43E−08	3.54E−03	4.72E−03
Pb	3.50E−03	3.00E−03	5.25E−04	2.47E−02	3.56E−07	5.67E−04	2.53E−02	1.44E−03	1.06E−07	2.88E−04	1.73E−03
Zn	3.00E−01	3.00E−01	6.00E−02	1.50E−03	1.86E−08	2.59E−05	1.53E−03	8.74E−05	5.53E−09	1.32E−05	1.01E−04
Cu	4.00E−02	4.00E−01	1.20E−02	3.50E−03	4.32E−09	4.02E−05	3.54E−03	2.03E−04	1.29E−09	2.04E−05	2.23E−04
Cr	3.00E−03	2.00E−04	6.00E−05	1.05E−02	1.95E−06	1.81E−03	1.23E−02	6.10E−04	5.79E−07	9.19E−04	1.53E−03
Ʃ	–	–	–	1.78E−01	5.95E−06	9.43E−03	**2.23E**−**01**	1.03E−02	1.77E−06	4.80E−03	**1.51E**−**02**

The carcinogenic risk to human health through exposure to heavy metals from wood pellet ashes was calculated for both children and adults as summarized in Table 8. If the Risk index is in the range from 1 × 10E−06 to 1 × 10E−04 the values were acceptable or tolerable for regulatory purposes^35^. The total Risk index calculated for exposure of children and adults to heavy metals from ash was 4.54E−05 and 3.21E−06, respectively (Table 8). Obtained results for total Risk index were lower than 1 × 10E−04 and they are generally considered acceptable for children and adults. Therefore, the carcinogenic risk caused by Ni, Co, Cd, Pb, and Cr in the ash could be negligible. Similar to HI values total Risk index values for children were also higher related to the values for adults, these results indicate that risk related to exposure to potentially polluted wood pellet ashes are higher for children than for adults.

**Table 8:** The cancer slope and Risk factors calculated for children and adults.

Element	SF_ing_	SF_inh_	Children			Adults		
			Risk_ing_	Risk_inh_	Risk_total_	Risk_ing_	Risk_inh_	Risk_total_
Ni	0.91	8.40E−01	2.88E−05	4.42E−10	2.88E−05	2.25E−06	1.31E−10	2.25E−06
Co	–	9.80E+00	–	9.13E−10	9.13E−10	–	2.72E−10	2.72E−10
Cd	–	6.30E+00	–	1.57E−09	1.57E−09	–	4.68E−10	4.68E−10
Pb	8.50E−03	4.20E−02	7.36E−07	4.49E−11	7.36E−07	4.28E−08	1.34E−11	4.28E−08
Cr	5.00E−01	4.10E+01	1.58E−05	1.60E−08	1.58E−05	9.16E−07	4.75E−09	9.21E−07
Ʃ	–	–	4.53E−05	1.90E−08	4.54E−05	3.21E−06	5.63E−09	3.21E−06

## Conclusion

This research has exposed the quantitative analysis of heavy metals in ten wood pellet ash samples. The health implications of these metals in the ash samples studied have also been identified. The results showed that the average concentrations of the heavy metals in the wood pellet ashes varied and decreased in the order Fe > Mn > Zn > Cu > Pb > Ni > Cr > Cd > Co. Heavy metal content of ash from wood pellets is a significant feature that allows an assessment of the behavior of these metals in the process of combustion and use of ash. The obtained concentration values of the analyzed metals are below the limits given by the law of individual European countries (European limit values).

The addition of ash to the soil is recommended to improve the chemical, physical and biological properties of the soil in agricultural production. However, values for MPI and EF indicate that long-term disposal of wood pellet ash can lead to soil contamination. Although this is the first study in B&H, the results obtained in this paper can serve as a basis for further monitoring. Hazard index (HI) for children and adults was lower than the safe limit indicating that there was no direct health risk from heavy metals from wood pellet ashes. Obtained results for the total risk index were lower than the limit value and they are generally considered acceptable for children and adults. Therefore, the carcinogenic risk caused by heavy metals in the ash could be negligible. In a view of all the metals, the results indicate that there is a low cancer risk. This study also has some limitations associated with the limited number of analyzed wood pellet samples, and our results are obtained on the laboratory production of ashes and may show some differences with those produced in home furnaces. The inclusion of a larger sample and survey data on the actual exposure to ashes from wood biomass is recommended.

## Data Availability

The data sets generated and/or analyzed during the current study are available from the corresponding author on reasonable request.
